# Therapeutic Approach of Chronic *Pseudomonas* Infection in Cystic Fibrosis—A Network Meta-Analysis

**DOI:** 10.3390/antibiotics10080936

**Published:** 2021-08-03

**Authors:** Orsolya Varannai, Noémi Gede, Márk Félix Juhász, Zsolt Szakács, Fanni Dembrovszky, Dávid Németh, Péter Hegyi, Andrea Párniczky

**Affiliations:** 1 Heim Pál National Peditric Institute, 1089 Budapest, Hungary; varannaiorsolya@gmail.com; 2 Institute for Translational Medicine, Medical School, University of Pécs, 7624 Pécs, Hungary; gede.noemi@gmail.com (N.G.); flixjuhsz@gmail.com (M.F.J.); szaki92@gmail.com (Z.S.); dembrovszky.f@gmail.com (F.D.); davidsum96@gmail.com (D.N.); hegyi2009@gmail.com (P.H.); 3 Doctoral School of Clinical Medicine, University of Szeged, 6720 Szeged, Hungary; 4 Centre for Translational Medicine, Semmelweis University, 1085 Budapest, Hungary; 5 Heart and Vascular Center, Division of Pancreatic Diseases, Semmelweis University, 1085 Budapest, Hungary

**Keywords:** FEV1, lung function decline, inhalative antibiotics

## Abstract

*****Pseudomonas* infection is a major determinant of morbidity and mortality in cystic fibrosis (CF). Maintaining optimal lung function in CF patients carrying *Pseudomonas* remains a challenge. Our study aims to investigate the efficacy of antipseudomonal inhaled antibiotics in CF patients with chronic *Pseudomonas* infection. A Bayesian network meta-analysis of randomized controlled trials was conducted. The main outcomes were changes in: (a) forced respiratory volume (FEV1), (b) *Pseudomonas aeruginosa* sputum density, and (c) CF Questionnaire Revised Respiratory Symptom Score (CFQR-RSS) at 4 weeks follow-up. Eighteen trials which reported on treatment with aztreonam lysine, tobramycin, colistin, levofloxacin, fosfomycin/tobramycin, and amikacin in various dosages were eligible for inclusion. In terms of change in FEV1%, aztreonam lysine (t.i.d., 75 mg) with a 28-day run in the tobramycin phase, aztreonam lysine (b.i.d., 75 mg) with a 28-day run in the tobramycin phase had the highest probability of being the most effective treatment (SUCRAs were 77, 76%, respectively). Regarding change in *Pseudomonas* sputum density, aztreonam lysine (b.i.d., 75 mg) with a 28-day run in the tobramycin phase, aztreonam lysine (t.i.d., 75 mg) with a 28-day run in the tobramycin phase had the highest probability of being the most effective treatment (SUCRAs were 90, 86%, respectively). Regarding change in CFQR-RSS, aztreonam lysine (t.i.d., 75 mg) and aztreonam lysine (b.i.d., 75 mg) with a 28-day run in the tobramycin inhalation solution phase had the highest probability of being the most effective treatments (SUCRA:74% and 72%, respectively). Regarding changes in FEV1% and *Pseudomonas* sputum density, aztreonam lysine with a run in tobramycin phase may be the best treatment option in treating chronic *Pseudomonas* in CF. According to CFQR-RSS no significant differences were found. Given the limitations of the studies included, validation trials are called for.

## 1. Introduction

Cystic fibrosis (CF) is a complex life-shortening genetic disorder [1]. It affects 1 out of 3000 Caucasian newborns [2]. The disease is caused by mutations in the cystic fibrosis transmembrane regulator (CFTR) gene encoding a chloride channel expressed in many epithelial and blood cells [3]. The life expectancy of patients with the disorder has been greatly increased over recent decades because of better notions of symptomatic treatment strategies [4].

The lungs, liver, pancreas, reproductive tract, sinuses, and secretory cells that express CFTR become dysfunctional in CF. A defect of mucociliar clearance, mucus plugging leads to secondary pulmonary infections, with *Pseudomonas aeruginosa* being the most common pathogen, followed by *Staphylococcus aureus, Burkholderia cepacia complex, Achromobacter xylosoxidans, Pandorea apista and Nontuberculous mycobacteria* [3,5]. Even though early and combined antibiotic therapies are available, they may fail to eliminate pathogens, thus leading to chronic colonization. By the age of 20, about 80% of CF patients become colonized and experience intermittent infection by *P. aeruginosa* [6]. Chronic *P. aeruginosa* infection (defined as >50% of the cultures being positive as per the Leeds criteria [7]) is complicated to manage and plays a major role in decreased lung function, as well as worsening mortality and morbidity rates [7,8]. When chronic infection develops, long-term inhaled antibacterial treatment is recommended to maintain lung function and prevent acute exacerbations that could lead to the progressive deterioration of lung function. In the event of failure of eradication therapy, long-term maintenance treatment is required with inhaled antibiotics [9]. Tobramycin, colistimethate sodium, aztreonam lysine, and levofloxacin are widely used antipseudomonal formulations. According to the CF Foundation and ECFS guidelines, chronic use of inhaled 2 × 300 mg tobramycin for 28 days of on/off therapy or aztreonam is recommended however treatment optimization is still in the focus of several further clinical studies [9]. Antibiotics should be initiated shortly after microbiological diagnosis to prevent injury, maintain lung function and to limit costs of hospitalizations and additional antibiotic use [10].

In recent decades, many randomized controlled trials have investigated chronic *Pseudomonas* infection in CF. Littlewood et al. [11] compared the efficacy and safety of inhaled antibiotics for chronic *Pseudomonas* infection, while Elborn et al. [12] investigated the efficacy of generally used inhaled antibiotics and levofloxacin inhalation solution in a network meta-analysis (NMA). NMA compares multiple treatments simultaneously in one analysis. It considers both direct comparisons of treatments within head-to-head RCTs and indirect comparison of treatments through a common comparator [13,14].

Since the latest NMA, papers on randomized controlled trials have been published and have provided further evidence in the field, warranting an update of evidence– most prominently regarding short-term efficacy: the number of eligible RCTs reporting on outcomes at 4 weeks have since doubled. Thus, our aim was to gather all available evidence on the short-term safety and efficacy off different antibiotic regimens in treating chronic *P. aeruginosa* infection, and to compare them to establish the best choice of therapy.

## 2. Results

### 2.1. Search, Selection, and Study Characteristics

Twenty-one RCTs were eligible for inclusion in the systematic review, 18 of which were included in the meta-analysis. All the included studies were randomized controlled trials (Table 1). The trials were published after 1990. The characteristics of the included trials are shown in Table 1.

### 2.2. Characteristics of Included Studies

The characteristics of participants and interventions are detailed in Table 1. All 18 trials had an almost equivalent rate of males and females. The studies involved two different age groups: 13 trials had a mean age of 20.1–35 years, and five involved a younger population (mean age: 11–19.5). All 18 studies declared that patients had microbiological evidence of chronic *P. aeruginosa* infection. The time elapsed between isolation of *P. aeruginosa* and allocation to treatment ranged from three to 12 months. 

Studies reported combinations of inhaled tobramycin, aztreonam lysine (AZLI), levofloxacin (LIS), amikacin (ALIS), fosfomycin/tobramycin (FTI), colistin (COL) and a placebo. As a treatment comparator of the four-week trial duration, six of the 18 studies involved tobramycin inhalation solution (TIS) and 13 trials included a placebo. AZLI was evaluated in five trials, LIS was in three, and COL and ALIS were in one. Three studies were not eligible for qualitative synthesis due to the overlapping patient population and incomparable unit of measurements of the outcomes [15,16,17]. 

### 2.3. Results of NMA

#### 2.3.1. Change in FEV1% Predicted from Baseline to Four Weeks 

**Figure 1:**
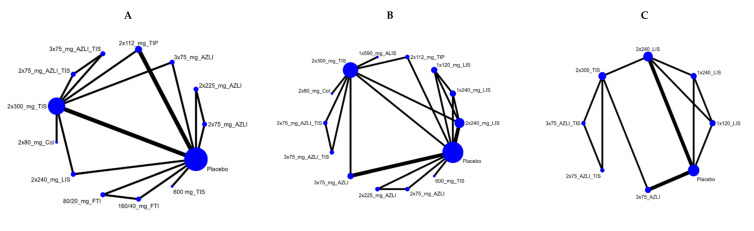
(**A**). Network graph of mean change in forced expiratory volume in 1 s per cent from baseline to four weeks. The widths of the lines are proportional to the number of studies, and the size of each circle is proportional to the sample size. TIS = tobramycin inhalation solution, AZLI = aztreonam lysine, FTI = fosfomycin/tobramycin, Col = colistin, LIS = levofloxacin. (**B**). Network graph of mean change in *Pseudomonas* sputum density from baseline to four weeks. TIS = tobramycin inhalation solution, AZLI = aztreonam lysine, TIP = tobramycin inhalation powder, Col = colistin, LIS = levofloxacin, ALIS = amikacin inhalation solution. (**C**). Network graph of change in CFQR-RSS, TIS = tobramycin inhalation solution, AZLI = aztreonam lysine, LIS = levofloxacin.

AZLI (t.i.d., 75 mg) with a 28-day run in the TIS phase, AZLI (b.i.d., 75 mg) with a 28-day run in the TIS phase and tobramycin inhalation powder (TIP) had the highest probability of being the most effective treatment options regarding FEV1% (SUCRA: 77%, 76% and 66%, respectively, see Figure S1. Since all the credible intervals included zero, the differences were statistically not significant in pairwise comparisons. Figure 1A shows the network graph, Figure S2 shows the cummulative ranking curves and Table S1 shows the league table and Figures S3–S9 show the forest plots for every possible comparison.

#### 2.3.2. Change in Pseudomonas Sputum Density 

**Figure 2:**
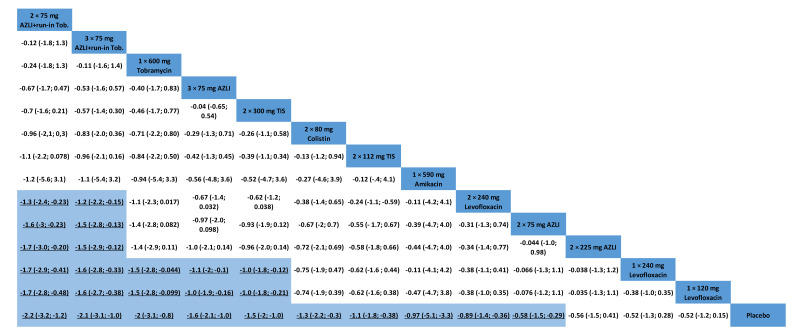
League table for change in *Pseudomonas* sputum density, log_10_ CFU/g sputum (credible intervals). Blue boxes show significant differences. TIS = tobramycin inhalation solution, AZLI = aztreonam lysine, TIP = tobramycin inhalation powder, Col = colistin, LIS = levofloxacin, ALIS = amikacin inhalation solution.

AZLI (b.i.d., 75 mg) with a 28-day run in the TIS phase, AZLI (t.i.d., 75 mg) with a 28-day run in the TIS phase and tobramycin (600 mg) had the highest probability of being the best treatments (SUCRA: 90%, 86% and 81%, respectively, see Figure S10). All the treatments proved to be more effective than placebo. In pairwise comparisons, since credible intervals did not include zero, these findings should be considered statistically significant. Figure 2 shows the league table and Figures S11–S23 show the forest plots for every possible comparison. Almost all treatments proved to be more effective than the placebo. Figure 1B shows the network graph, Figure S24 shows the cummulative ranking curves.

#### 2.3.3. Change in CFQR-RSS

Five trials reported on the change in CFQR-RSS from baseline to four weeks. AZLI (t.i.d., 75 mg) and AZLI (b.i.d., 75 mg) with a 28-day run in the TIS phase had the highest probability of being the best treatments (SUCRA: 74% and 72%, respectively, see Figure S25). We did not find significant differences. Figure 1C shows the network graph, Figure S26 shows the cummulative ranking curves, Table S2 shows the league table and Figures S27–S32 show the forest plots for every possible comparison.

#### 2.3.4. Adverse Events

Adverse events are presented in Table 1. Safety was documented in 17 RCTs, although they failed to distinguish between the drug-related events or symptoms caused by the deterioration of CF. These imprecise definitions made it difficult to evaluate the adverse event, especially in the presence of coughing, which was the most frequently reported adverse event. The adverse events reported by patients were mostly coughing, productive coughing, a decrease of FEV1% and hemoptysis.

#### 2.3.5. Risk-of-Bias Assessment and Quality of Evidence

Seventeen studies were judged to raise some concerns about change in FEV1 outcome. Sixteen studies were considered to raise some concerns about change in *Pseudomonas* sputum density. Further information can be found in the risk-of-bias tables and in the graphic risk-of-bias summary in the (supplementary material Figures S10–S11). The quality of evidence was rated as very low for ten comparisons and moderate for two comparisons regarding change in *Pseudomonas* sputum density (detailed in Table S3) and was very low for all comparisons regarding change in FEV1% (detailed in Table S4).

**Table 1:** Study characteristics, Tin = tinnitus, Hpt = hemoptysis, C = coughing, AE = any adverse event, NA = not applicable (*incomparable unit of measurement), ND = no data.

Authors (Year of Publication)	Countries and No. of Centers	Inclusion Period	Intervention/Comparator	Mean Age (Years)	% of Females	No of Randomized Patients	Study Duration (Weeks)	Adverse Events	Treatment Schedule	Baseline FEV1%	FEV1% Change at 4 Weeks
Ramsey et al. (1993) [18]	USA, 7 centers	March 1989–June 1991	3 × 600 mg tobramycin	17.7	42%	71	24	Not reported	Intervention 3 times a day or comparator	55%	3.72%
			Placebo	16.6	54%					60%	−5.97%
Ramsey et al. (1999) [19]	USA, 69 centers	August 1995–October 1996	2 × 300 mg tobramycin inhalation solution	20.8	42%	520	24	Tin: 3.1% Hpt: 26.7%	3 cycles of 28 days on treatment and 28 days off treatment for a total of 24 weeks	49.9%	11.98%
			Placebo	20.6	50%			Tin: 0% Hpt: 30.9%		51.2%	0.057%
Hodson et al. (2002) [20]	UK, Ireland, 16 centers	Not reported	2 × 300 mg tobramycin inhalation solution	21.3	62.3%	126	8	AE: 64.2% c: 9.4%	Intervention twice a day or comparator for 4 weeks and 4 weeks of follow-up	55.4%	6.7%
			2 × 80 mg colistin sulfomethate	20.1	48.4%			AE: 50.0%c: 17.7%		59.4%	0.37%
Lenoir et al. (2007) [21]	France, Italy, Ukraine, Moldova, 13 centers	Not reported	2 × 300 mg tobramycin inhalation solution	11	48.3%	59	8	Drug-related: 10.3%	Intervention twice a day or comparator for 4 weeks and 4 weeks of follow-up	57.7%	16.11%
			Placebo	14.2	44.3%			23.3%		59.8%	2.53%
Chuchalin et al. (2007) [22]	Hungary, Poland, Russia, 21 centers	Not reported	2 × 300 mg tobramycin inhalation solution	14.8	44.7%	247	24	Drug-related: AE: 15.5% c (not detailed): 2.2%	3 cycles of 28 days on treatment and 28 days off treatment for a total of 24 weeks	60.7%	7.81%
			Placebo	14.7	45.2%			AE: 15.3% c (not detailed): 69.4%		63.6%	0.55%
Retsch-Bogart et al. (2008) [23]	USA, 20 centers	November 2003–August 2004	2 × 75 mg aztreonam lysine	27.2	43.2%	105	5	Drug-related: AE: 27% c: 13.5% AE: 37.8% c: 18.9% AE: 19.4% c: 9.7%	Twice a day for 14 days	74.27%	0.59%
			2 × 225 mg aztreonam lysine	23.9	48.6%					81.23%	0.86%
			Placebo	27	54.8%					76.84%	0.49%
McCoy et al. (2008) [24]	USA, 56 centers	February 2005–September 2006	2 × 75 mg aztreonam lysine	26.5	44.9%	246	12	(not drug-related) c: 27.5%	28-day run in tobramycin phase. 4 weeks of intervention or comparator and 8-week follow-up phase	56.3%	3.78%
			3 × 75 mg aztreonam lysine	24.1	42.4%			36.4%		55.4%	4.09%
			Placebo	27.9	40.8%			34.2%		53.9%	−2.42%
Retsch-Bogart et al. (2009) [25]	USA, Canada, Australia, New Zealand, 53 centers	June 2005–April 2007	3 × 75 mg aztreonam lysine	27.4	40%	164	6	c (not drug-related): 35%	Intervention for 28 days or comparator 3 times a day	NA *	NA *
			Placebo	31.7	46.4%			29.8%			
Wainwright et al. (2011) [26]	Australia, Canada, USA, 40 centers	June 2008–June 2009	3 × 75 mg aztreonam lysine	19.5	39.5%	157	6	c related to study drug: 9.2%	Intervention for 28 days or comparator 3 times a day	95.5%	0.29%
			Placebo	18.9	45.7%			4.9%		94.7%	−2.5%
Konstan et al. (2010) [27]	15 countries, 127 centers	Not reported	2 × 112 mg tobramycin inhalation powder	25	45%	517	24	c related to study drug: 25.3% 4.3%	3 cycles of 28 days on treatment and 28 days off treatment for a total of 24 weeks	53%	2.76%
			2 × 300 mg tobramycin inhalation solution	26	44.5%					53%	3.55%
Konstan et al. (2011) [28]	Bulgaria, Lithuania, Serbia, Argentina, Brazil, Chile, Mexico, USA, 38 centers	September 2005–February 2007	2 × 112 mg tobramycin inhalation powder	13.4	58.7%	93	4 + 12	c (not drug-related): 13%	3 cycles of 28 days on intervention or comparator and 2 cycles of 28 days on treatment for a total of 24 weeks	54.7%	12.96%
			Placebo	13.2	53.1%			26.5%		58.5%	−0.59%
Geller et al. (2011) [29]	USA, Europe, 51 centers	June 2008–June 2009	1 × 120 mg levofloxacin inhalation solution	28	47.4%	151	8	c (not detailed): 15.8%	28 days on treatment and 28 days off treatment, 1 cycle	52.9%	
			1 × 240 mg levofloxacin inhalation solution	27.5	43.2%			16.2%		55.4%	
			2 × 240 mg levofloxacin inhalation solution	29.2	35.9%			15.4%		48.8%	
			Placebo	30.1	48.6%			10.8%		52.4%	
Trapnel et al. (2012) [30]	USA, 33 centers	June 2008–January 2010	80/20 mg fosfomycin/tobramycin for inhalation	35	45%	119	8	Related to study drug AE: 29% c: 10%	28 days of treatment	50%	1%
			160/40 mg fosfomycin/tobramycin for inhalation	31	51%			AE: 51% c: 7%		48%	−0.3%
			Placebo	31	43%			AE: 15% c: 10%		48%	−6.5%
Galeva et al. (2013) [31]	Bulgaria, Estonia, Latvia, Lithuania, 17 centers	June 2009–May 2011	2 × 112 mg tobramycin inhalation powder	12.9	70%	62	8	Drug-related AE: 16.7%	28 days on treatment and 28 days off treatment	59.1%	8.2%
			Placebo	12.9	59.4%			6.3%		59.3%	2.3%
Assael et al. (2013) [32]	Europe, USA, 91 centers	August 2008–May 2010	3 × 75 mg aztreonam lysine	25.8	50%	273	24	Drug-related AE: 22.8% c (not drug-related): 70.6%	3 cycles of 28 days on intervention or comparator	52.3%	8.064%
			2 × 300 mg tobramycin inhalation solution	25.1	50%			AE: 12.9% c (not drug-related): 78.8%		52.2%	−0.14%
Elborn et al. (2015) [33]	Europe, USA, Israel, 125 centers	February 2011–August 2012	2 × 240 mg levofloxacin inhalation solution	28.1	45.5%	282	24	c (not drug-related): 53.3%	3 cycles of 28 days on intervention or comparator	54.8%	2.33%
			2 × 300 mg tobramycin inhalation solution	28.8	39.8%			58.2%		53.2%	0.42%
Flume et al. (2016) [34]	USA, Canada, Australia, New Zealand, Israel, 97 centers	October 2010–May 2012	2 × 240 mg levofloxacin inhalation solution	29.4	47.9%	330	8	Drug-related AE: 27.9%	28 days on treatment and 28 days off treatment	56.6%	1.73%
			Placebo	28.8	42.7%			18.2%		56.3%	0.411%
Bilton et al. (2019) [35]	Europe, Canada, 70 centers	February 2012–September 2013	1 × 590 mg amikacin liposome inhalation suspension	22.8	46.6%	302	24	Drug-related AE: 38.5 c: 8.8%	3 cycles of 28 days on intervention or comparator	64.5%	4.14%
			2 × 300 mg tobramycin inhalation solution	22	47.9%			14.4 c: 2.1%		61.9%	7

## 3. Discussion

The aim of this study was to rank all the available inhaled antibiotics by efficacy represented by improvement in FEV1% and CFQR-RSS and by the reduction of *Pseudomonas* sputum density after four weeks of treatment. A total of 2 × 75 mg AZLI with 28 days of TIS pre-treatment ranked the highest and was significantly more effective than a placebo and five other antibiotic formulations in reducing *Pseudomonas* sputum density. Although 3 × 75 mg AZLI combined with 28 days of TIS ranked the highest as regards improvement of FEV1%, we failed to detect any significant difference between the antibiotic formulations in pairwise comparisons. In contrast, 3 × 75 mg AZLI ranked the highest for CFQR-RSS, without significant differences across groups in pairwise comparisons. 

Compared to previous NMAs conducted by Littlewood et al. [13] and Elborn et al. [12] our study includes recent publications and trials not involved in previous work detailed in Table 2. In 2012, Littlewood et al. included 11 trials comparing five antibacterial agents with their results suggesting that all regimens lead to improvement compared to a placebo, although there were no significant differences to support their findings. In 2016, Elborn et al. included LIS as a new formulation for treatment, and they compared the efficacy of the most widely used inhaled antibiotics. They found that after four weeks of treatment, LIS is significantly more effective in reducing *Pseudomonas* sputum density than a placebo. However, tobramycin formulations and AZLI improved sputum density numerically more effectively after four weeks and 24 weeks of therapy. The novelty of our NMA is the investigation of different dosages in analysis and the addition of ALIS as a new antipseudomonal antibiotic to the network.

In accordance with the previous guidelines in chronic *Pseudomonas* infection, long-term tobramycin inhalation solution is the first choice of treatment and tobramycin inhalation powder seems to be equivalently effective. As an alternative treatment, AZLI is approved by European [9] and USA guidelines [10]. The European guideline recommends 300 mg TIS twice a day, while our results show that a combination of TIS and AZLI is more effective than TIS alone. 

One advantage of AZLI is the shorter duration of inhalation as it requires 2–3 min for nebulization compared to tobramycin inhalation, which takes about 15–20 min. The shorter administration period could improve patient compliance [36]. On the other hand, twice daily inhalation could be more convenient than three times. 

Regarding side-effects of TIS, systematic toxicity is relatively rare; however, tinnitus and hoarseness were reported, and long-term use were associated with ototoxicity and nephrotoxicity [36]. With tobramycin inhalation solution, voice alteration (12.8%), myalgia (4.7%) and laryngitis (4.3%) are the most common side-effects. Regarding aztreonam inhalation, coughing (54%), nasal congestion (16%) and wheezing (16%) are the most common adverse effects [37]. In 2017, the annual cost of TIS was approx. USD 19,056 compared to AZLI, whose annual cost was USD 26,366 [38]. Of note, AZLI preparation is not licensed for use with patients under the age of 6 years and is not available in many European countries such as Hungary.

The NMA had some limitations. Previous treatment with the active drug might have affected the outcomes in some studies, while there were trials where the population was naive to the active agent. Those who had not been exposed earlier might have achieved a more noticeable improvement than those who had already been affected by the active drug. The results of our NMA should be interpreted carefully because of the clinical heterogeneity in the trials included, although this is not so significant as to cause intransitivity. The withdrawal rate of an open-label study may be affected by the physician or the patient. The included studies were heterogeneous in the overall disease severity of their included patients. Those who had very low lung function values and severe pulmonary involvement at the beginning of the trial improved more than those whose baseline FEV1 values were higher. Two types of chronological bias could have occurred. The NMA also has a higher risk of chronological bias as the earliest trial that we were able to include was from 1993 and the most recent trial was from 2019. All the studies come from developed countries, where accessibility of different antibiotics is unrestricted. In the past 20 years, the *Pseudomonas* resistance profile could have changed, and the bacterium might have been susceptible to other potential agents, although trials did not report this problem and did not assess the resistance profile. Nevertheless, antibiotic resistance could differ in various parts of the world, and the included studies came from different countries and continents. The USA, Western Europe, and Australia were overrepresented which could influence transitivity. Child and adult subpopulations were not examined due to the insufficient data.

One strength is that the inconsistency test with node splitting did not suggest any inconsistencies for any outcomes, which is the reason clinical heterogeneity does not cause intransitivity as detailed in Figures S33–S35 The small-study effect is unlikely to distort our results, as indicated by the funnel plots detailed in Figure S36. Some of the studies were open-label, which has a higher risk of performance or detection bias.

## 4. Materials and Methods

### 4.1. Search Strategy 

**Figure 3:**
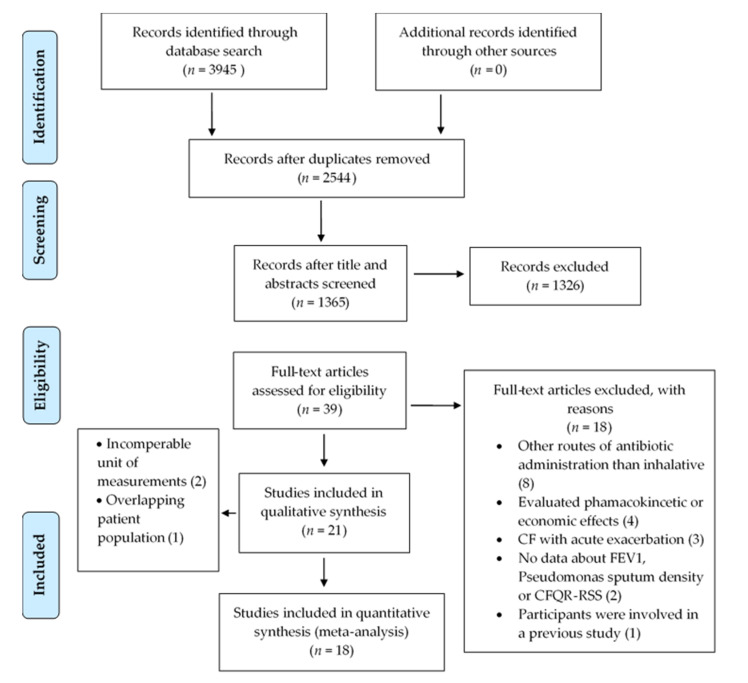
PRISMA flow chart.

This NMA is reported based on the Preferred Reporting Items for Systematic Reviews and Network Meta-Analysis statement (PRISMA) [39]. The NMA entailed a systematic search in CENTRAL, MEDLINE (via PubMed), Scopus, Web of Science, and Embase from the date of database inception to 7 October 2019, using free-text terms as follows: (((cystic fibrosis OR mucoviscidosis OR ‘lung disease’ OR ‘genetic disease’ OR cftr))) AND ((*Pseudomonas* OR P. aeruginosa)) AND Random*. We placed no restrictions such as year of publication or language on the search. Each document was downloaded into the EndNote X9 citation manager (Clarivate Analytics, Philadelphia, PA, USA) and title duplicates were eliminated by the citation manager and then manually detailed in Figure 3.

### 4.2. Selection and Eligibility 

For our clinical question, we used the PICO form. The population was CF patients chronically infected with *Pseudomonas aeruginosa*. The intervention and comparators were different inhalative antipseudomonal agents. The primary outcome was change in forced expiratory volume in 1 s % (FEV1%); the secondary outcomes were change in *Pseudomonas* sputum density and change in cystic fibrosis questionnaire revised respiratory symptom scores (CFQR-RSS).

Included participants were CF patients over six years of age chronically infected with *Pseudomonas aeruginosa*. We accepted the CF diagnostic criteria and the definition of chronic infection used by the individual articles. However, the definitions used were mostly consistent we noted no clinically relevant differences. Therapy with inhalative antipseudomonal agents for a duration of 4 to 24 weeks were accepted, studies including children or adults with a clinical diagnosis and positive sweat or genetic test of CF with chronic *Pseudomonas* infection were eligible. Disease severity was different in all the trials.

We examined the mean change in FEV1% from baseline to four weeks as the primary outcome. The secondary outcomes were mean change in *P. aeruginosa* sputum density from baseline to four weeks of therapy and mean change in the CFQR-RSS from baseline to four weeks. The questionnaire is a widely used, validated, patient-reported, disease-specific instrument in adolescents and adults for measuring quality of life (QoL). It contains 14 items on 12 generic and disease-specific scales, with scores ranging from 0 to 100 in each domain and higher scores indicating better health-related QoL [40]. All randomized controlled trials fitting our PICO were eligible for inclusion in this systematic review.

### 4.3. Data Extraction

In accordance with the inclusion criteria, two independent investigators (OV, MFJ) screened the titles and abstracts to choose eligible full-text articles. Relevant information was extracted from the included studies to a predesigned data form, including author, year of publication, baseline characteristics, type of intervention, dosage, baseline values, and post treatment values of the outcomes. Data were collected by the first author (OV). The second author (MFJ) reviewed the datasheet. Disagreements in the selection and data-collection were resolved by a third review author (FD).

### 4.4. Statistical Analysis

A Bayesian method was used to conduct pairwise meta-analyses and an NMA. All the analyses were carried out with a random effect model. Mean difference (MD) was calculated for the continuous outcomes with 95% credible intervals (95%CrI). We optimized the model and generated posterior samples using Markov Chain Monte Carlo methods running in four chains. We set at least 20,000 adaptation iterations to achieve convergence and 10,000 simulation iterations. We also ranked interventions by their posterior probability by calculating the Surface Under the Cumulative Ranking (SUCRA) curve values. Inconsistency was evaluated by node splitting. Funnel plots were created for each outcome and Egger’s tests were conducted to assess the small-study effect, Figure S36. Deviation information was expressed for outcome results, such as 95% confidence intervals and standard error, to evaluate missing standard deviations. All computations were done using R (V. 3.5.2, “*R & R*” of the Statistics Department of the University of Auckland) package gemtc (V. 0.8-2) along with the Markov Chain Monte Carlo engine JAGS (V. 3.4.0), package netmeta (V. 1.1-0) and STATA 16.0 (StataCorp LLC, 4905 Lakeway Drive College Station, TX 77845-4512, USA). 

### 4.5. Risk-of-Bias Assessment and Quality of Evidence

Risk of bias was assessed by the RoB 2 Cochrane risk-of-bias tool [39] for randomized trials recommended by the Cochrane Collaboration [41]. Two review authors (DN, FD) assessed the risk of bias independently, detailed in Supplementary material, Figures S37 and S38. Where conflict arose, a third person made the final decision (ZS). We used the Grading of Recommendations Assessment, Development, and Evaluation (GRADE) approach to assess the quality of evidence [42].

### 4.6. Protocol Registration

This study protocol was registered with PROSPERO under registration number CRD42020160311. We deviated from the protocol in a single matter: we decided to include CFQR-RSS in our examined outcome parameters—this was not among our initial interests, but we decided that it should be included since it is a patient important outcome that we could appropriately address in our work. Deviations from the PROSPERO protocol were that there were not enough new RCTs to construct a network investigating hospitalization rate and time to pulmonary exacerbation.

## 5. Conclusions

The results of this NMA showed that aztreonam lysine in combination with tobramycin inhalation solution is the best available choice in treating chronic *Pseudomonas* infection in CF. Even if these antibiotics are not licensed in all European countries, the high costs of this antibiotic therapy may outweigh the long-term reduction in cost of care as improvement in nutritional and pulmonary status results in better life expectancies. In the future, further randomized controlled trials are needed to evaluate antibiotics and their effects on acute and chronic infections. Novel antimicrobial agents or combination of therapies should also be investigated, to improve treatment of bacterial infections. This article highlights the synergistic antimicrobial activity of gene modulator therapies and antimicrobial treatments aztreonam lysin should also be studied in this aspect in the future [43]. Traditional therapeutic strategies for the treatment of chronic *Pseudomonas* infection in CF patients stand on the administration of single inhaled antibiotic formulation [44]. Despite several clinical trials have shown the efficacy of rigorous antibiotic treatment [45], eradication fails in approximately 10–40% of CF patients with chronic *P. aeruginosa* infection [46,47] Therefore, several basic and clinical research focus on possible new therapeutic approaches [48,49], antagonistic interaction seems to be one of them [50]. In accordance with our results, the sequential regime leads to hypersusceptibility to antibiotics and reduce the amount of resistant mutants [51]. 

Given the limitations of the studies included, validation trials are called for. Currently, there is no ongoing trial which investigates the treatment of chronic *Pseudomonas* infection.

## Figures and Tables

**Table 2:** Comparison of previous NMAs.

	Littlewood et al. 2012 [11]		Elborn et al. 2016 [12]		Current NMA “Varannai et al. 2021”	
Duration	4 weeks	20 weeks	4 weeks	24 weeks	4 weeks	24 weeks
No. of RCTs	7 trials	Insufficient data	7 trials	9 trials	FEV1: 14 trials, CFQ: 7 trials Pa.: 14 trials	9 trials
Antibiotics involved	TIS (300 mg/4 mL), TIS (300 mg/5 mL), TIP, colistin, AZLI		TIS, TIP, colistin, AZLI, LIS	TIS, TIP, AZLI, LIS	TIS, TIP, colistin, AZLI, LIS, amikacin, FTI	TIS, TIP, AZLI, LIS
Outcome	FEV1, Pa. sputum density, acute exacerbations		FEV1 relative and absolute change, Pa. sputum density, CFQR-RSS change, hospitalization, use of additional antibiotics, study withdrawal rates.		Relative change in FEV1%, change in *Pseudomonas* sputum density, change in CFQR-RSS, hospitalization, time to acute exacerbation.	
Results	All treatments led to an improvement compared to a placebo; tobramycin formulations led to an improvement over AZLI or colistin (although these were not significant differences).		The relative change in FEV1% was numerically the highest with AZLI. LIS reduced *Pseudomonas* sputum density significantly better than a placebo, although TIP, TIS, and AZLI were numerically more effective. As regards hospitalization, an indirect comparison was conducted due to a lack of trials. Additional antibiotics were required for TIS- and placebo-treated patients compared to LIS.	Changes in FEV1 and sputum density were numerically more effective with LIS compared with TIP and TIS, and they were significantly better than with a placebo. Significantly fewer patients were hospitalized with LIS than with TIP, TIS, and a placebo. Additional antibiotics were required for TIS-, TIP-, and placebo-treated patients compared to LIS.	Aztreonam combined with 28 days of tobramycin were the best treatments as regards changes in FEV1% and sputum density.	The NMA was not conducted for 24 weeks to avoid reproduction of the result from the previous NMA.

## Data Availability

The data presented in this study are available on request from the corresponding author.

## Supplementary Materials

The following are available online at https://www.mdpi.com/article/10.3390/antibiotics10080936/s1, Figure S1: Surface under the cumulative ranking curves (SUCRA%) values of chane in FEV1%; AZLI= aztreonam lysine, TIS = tobramyin inhalation solution, TIP = tobramycin inhalation powder, LIS = levofloxacin inhalation solution, FTI = fosfomycin/tobramycin, Figure S2: Cumulative ranking curves of change in FEV1%, Figures S3–S9: Forest plots for change in FEV1%., Figure S10 Surface under the cumulative ranking curves (SUCRA%) values of change in Pseudomonas sputum density; AZLI= aztreonam lysine, TIS = tobramyin inhalation solution, TIP = tobramycin inhalation powder, LIS = levofloxacin inhalation solution, ALIS: amikacin inhalation solution, Figures S11–S23: Forest plots for change in Pseudomonas sputum density. Figure S24 Cumulative ranking curves of change in Pseudomonas sputum density, Figure S25 Surface under the cumulative ranking curves (SUCRA%) values of change in CFQR-RSS. Figure S26 Cumulative ranking curves of change in CFQR-RSS. Figures S27–S32: Forest plots for change in CFQR-RSS. Figures S33–35: Consistency test. Figure S36: Funnel plots and Egger’s tests. Figures S37 and S38: Summary of risk of bias assessment. Table S1: League table of change in FEV1%. The overall risk of bias assessment were judged to raise some concern and in line with the GRADE approach all comparisons were judged as very low quality ⊕◯◯◯. Table S2: League table of change in CFQR-RSS, Table S3: summary of findings of change in FEV1%, Table S4: summary of findings of change in Pseudomonas sputum density.
