# Supplementary material for: Therapeutic Approach of Chronic Pseudomonas Infection in Cystic Fibrosis—A Network Meta-Analysis
Source: Antibiotics (Basel). 2021 Aug 3;10(8):936. doi: 10.3390/antibiotics10080936 (PMC8388982; doi:10.3390/antibiotics10080936)
Supplement: Supplementary file 1 [file antibiotics-10-00936-s001.zip › antibiotics-1275175-supplementary.pdf]

antibiotics

MDPI

Therapeutic Approach of Chronic *Pseudomonas* Infection in Cystic Fibrosis—A Network Meta-Analysis

Orsolya Varannai [1,2,3] Noémi Gede [2], Márk Félix Juhász [2], Zsolt Szakács [2], Fanni Dembrovszky [2], Dávid Németh [2], Péter Hegyi [2,4,5] and Andrea Párniczky [1,2,4,*]

1 Heim Pál National Peditric Institute, 1089 Budapest, Hungary; varannaiorsolya@gmail.com
2 Institute for Translational Medicine, Medical School, University of Pécs, 7624 Pécs, Hungary; gede.noemi@gmail.com (N.G.); flixjuhsz@gmail.com (M.F.J.); szaki92@gmail.com (Z.S.); dembrovszky.f@gmail.com (F.D.); davidsum96@gmail.com (D.N.); hegyi2009@gmail.com (P.H.)
3 Doctoral School of Clinical Medicine, University of Szeged, 6720 Szeged, Hungary
4 Centre for Translational Medicine, Semmelweis University, 1085 Budapest, Hungary
5 Division of Pancreatic Diseases, Heart and Vascular Center, Semmelweis University, 1085 Budapest, Hungary
* Correspondence: andrea.parniczky@gmail.com

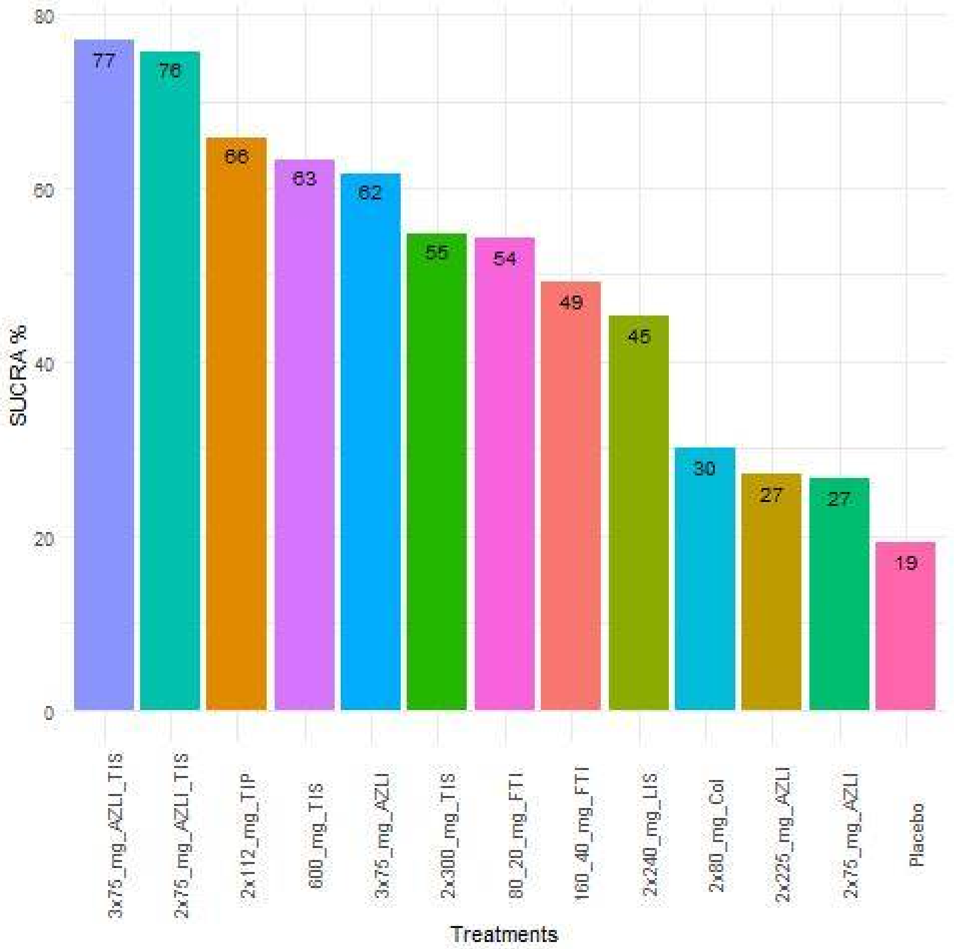

Figure S1. Surface under the cumulative ranking curves (SUCRA%) values of chane in FEV1%; AZLI= Aztreonam lysine, TIS=Tobramyin inhalation solution, TIP=Tobramycin inhalation powder, LIS=Levofloxacin inhalation solution, FTI=Fosfomycin/Tobramycin.

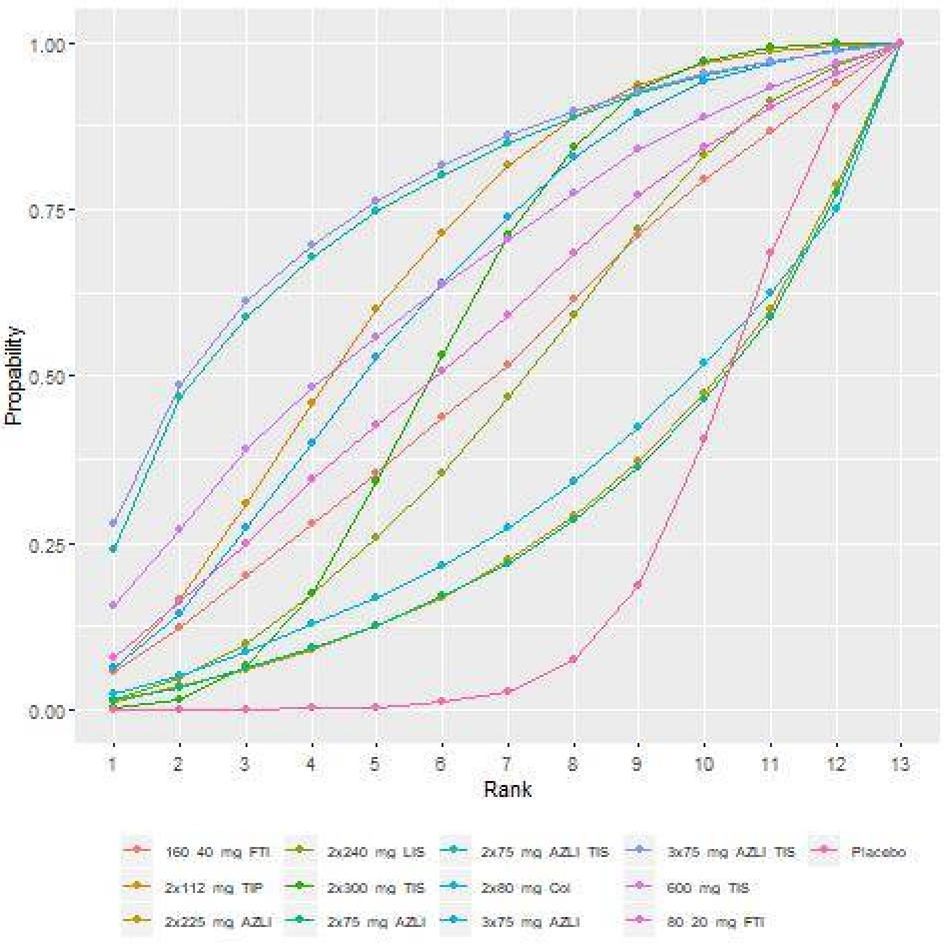

Figure S2. Cumulative ranking curves of change in FEV1%.

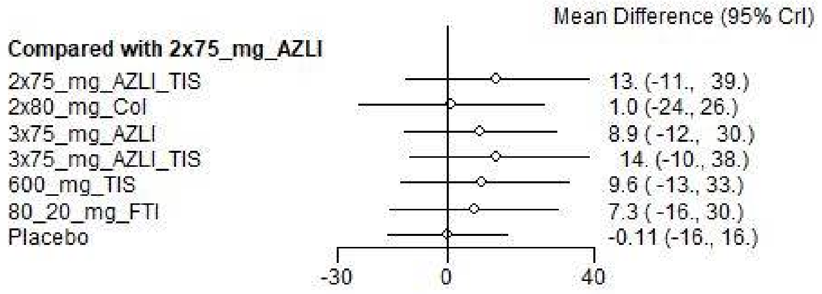

Figure S3. Forest plot for change in FEV1%, interventions compared to 75 mg AZLI (b.i.d.).

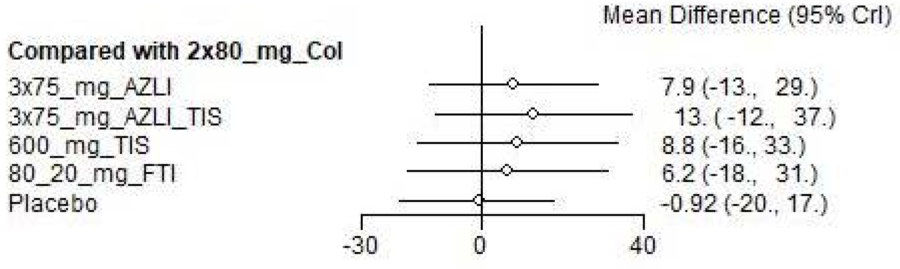

Figure S4. Forest plot for change in FEV1%, intervention compared to 80 mg Colistin (b.i.d.).

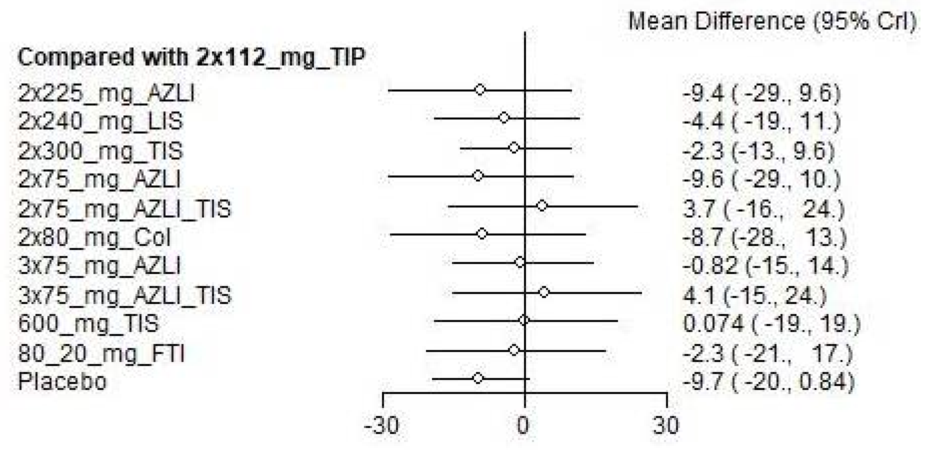

Figure S5. Forest plot for change in FEV1%, intervention compared to 112 mg Tobramxin inhalation powder (b.i.d.).

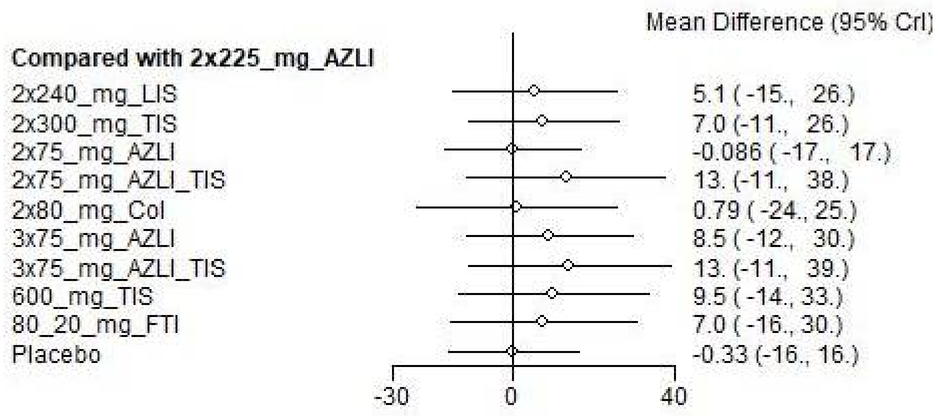

Figure S6. Forest plot for change in FEV1%, intervention compared to 225 mg Aztreonam lysine (b.i.d.).

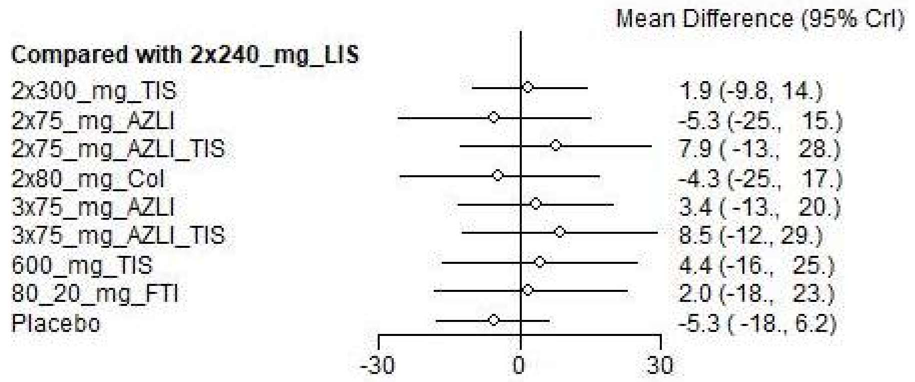

Figure S7. Forest plot for change in FEV1%, intervention compared to 240 mg Levofloxacin inhalation solution (b.i.d.).

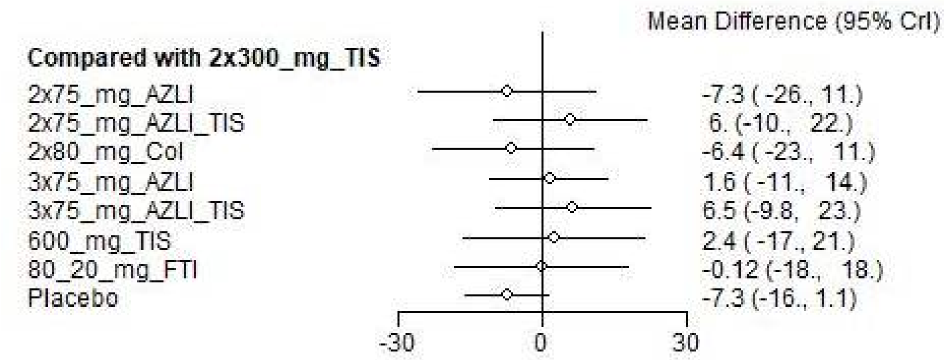

Figure S8. Forest plot for change in FEV1%, intervention compared to 300 mg Tobramycin inhalation solution (b.i.d.).

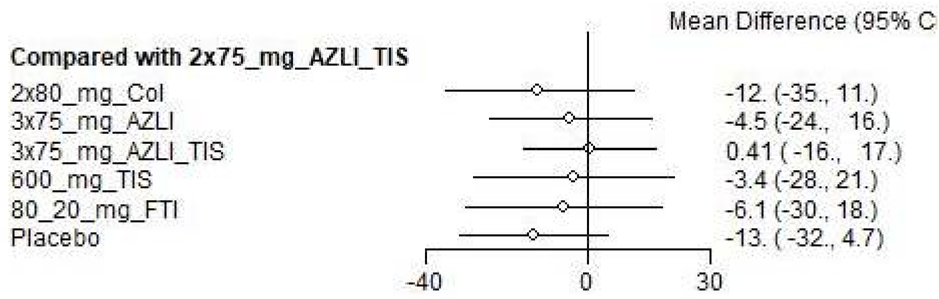

Figure S9. Forest plot for change in FEV1%, intervention compared to 75 mg Aztreonam lysine with run in Tobramycin.

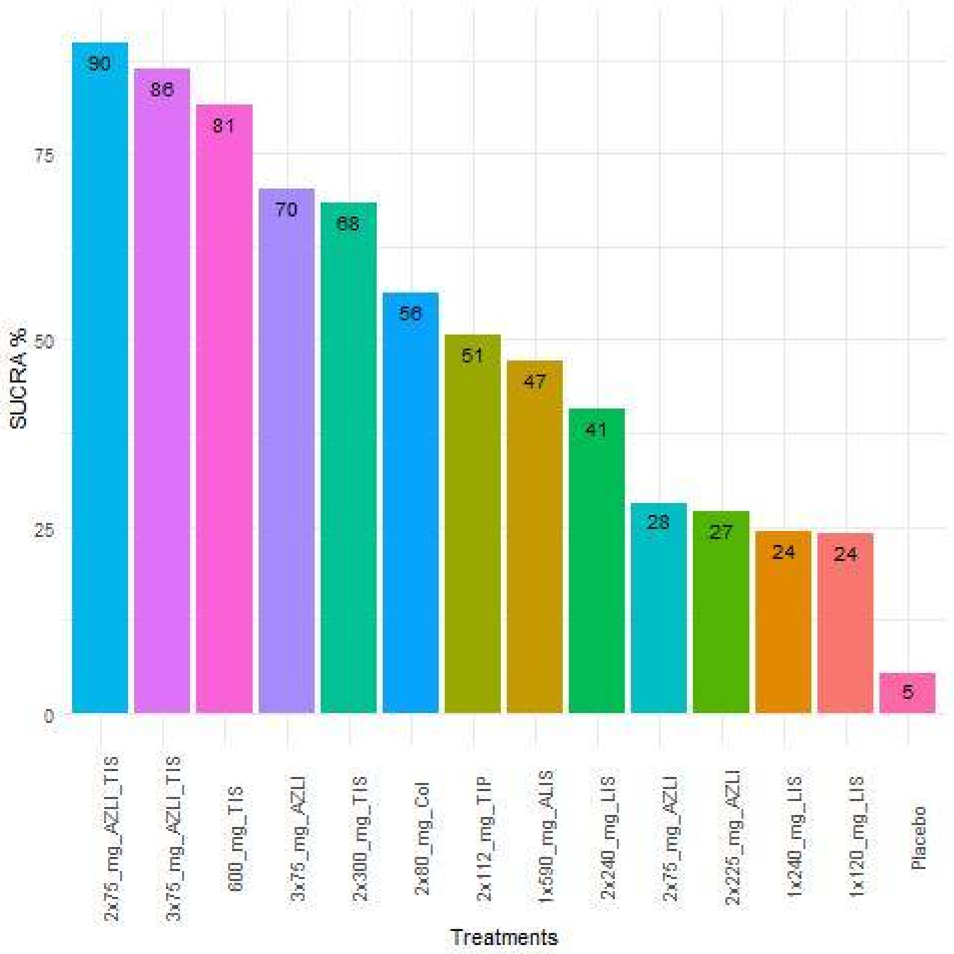

Figure S10. Surface under the cumulative ranking curves (SUCRA%) values of change in Pseudomonas sputum density; AZLI= aztreonam lysine, TIS=tobramyin inhalation solution, TIP=tobramycin inhalation powder, LIS=levofloxacin inhalation solution, ALIS: amikacin inhalation solution.

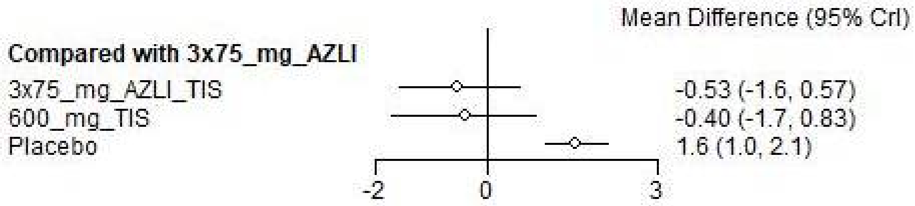

Figure S11. Forest plot for change in Pseudomonas sputum density interventions compared to 3 × 75 mg aztronam.

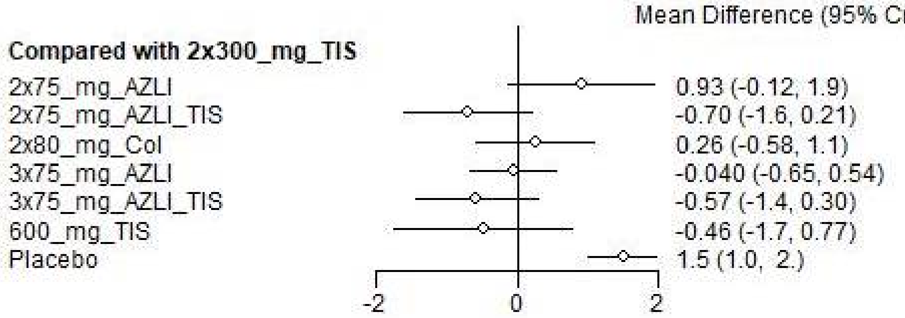

Figure S12. Forest plot for change in Pseudomonas sputum density interventions compared to 2 × 300 mg tobramycin inhalation solution.

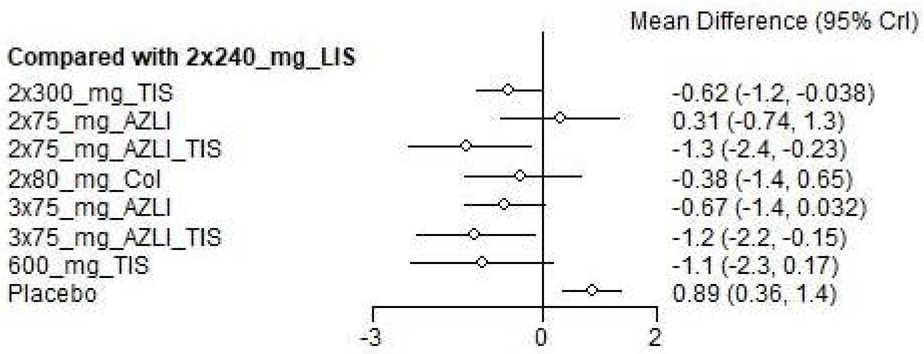

Figure S13. Forest plot for change in Pseudomonas sputum density interventions compared to 2 × 240 levofloxacin inhalation solution.

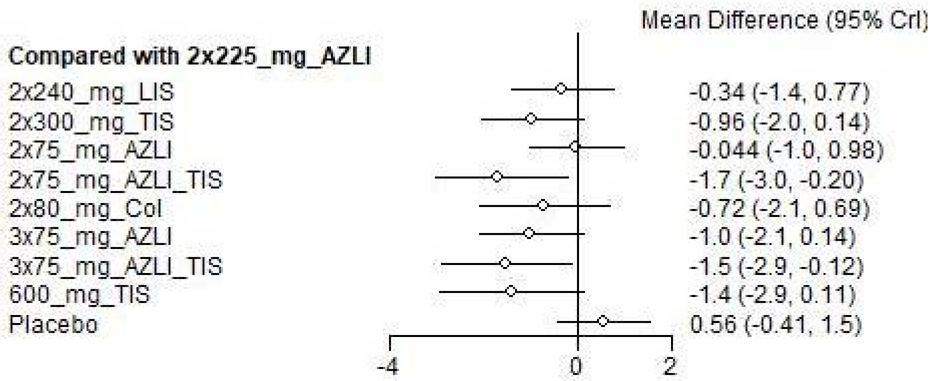

Figure S14. Forest plot for change in Pseudomonas sputum density interventions compared to 2 × 225 mg aztronam-lysine.

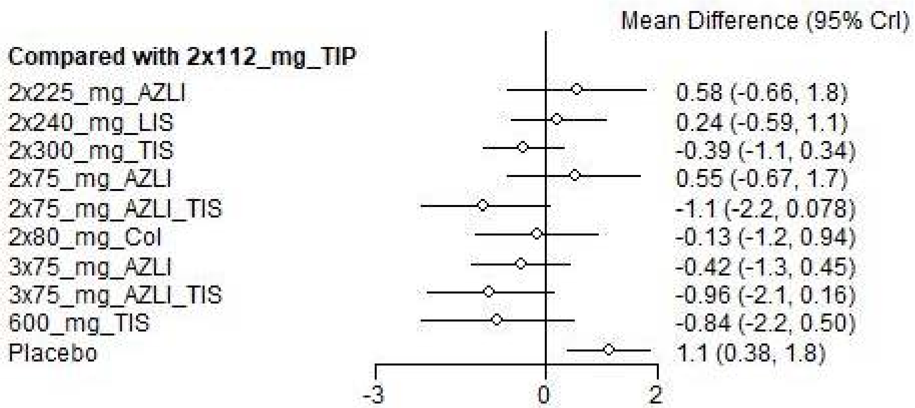

Figure S15. Forest plot for change in Pseudomonas sputum density interventions compared to 2 × 112 mg tobramycin inhalation powder.

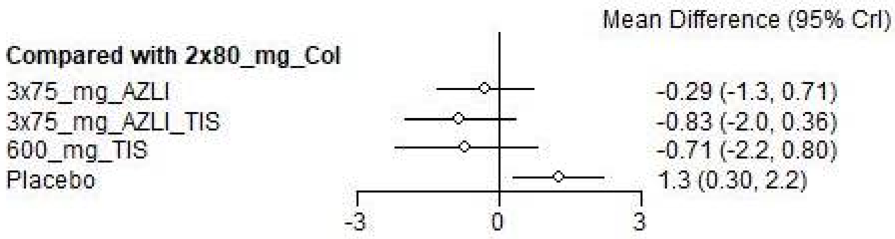

Figure S16. Forest plot for change in Pseudomonas sputum density interventions compared to 2 × 80 mg colistin.

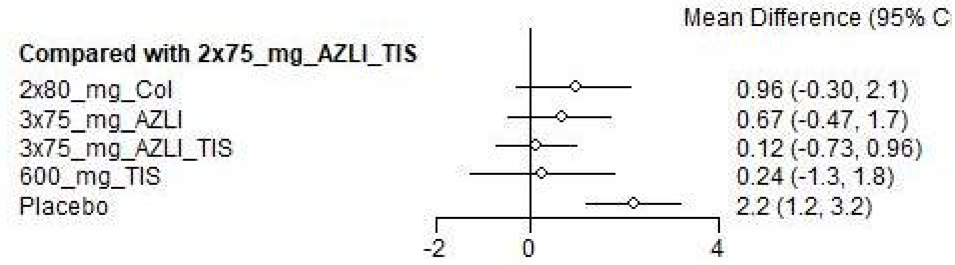

Figure S17. Forest plot for change in Pseudomonas sputum density interventions compared to 2 × 75 mg aztronam-lysine+run in tobramycin.

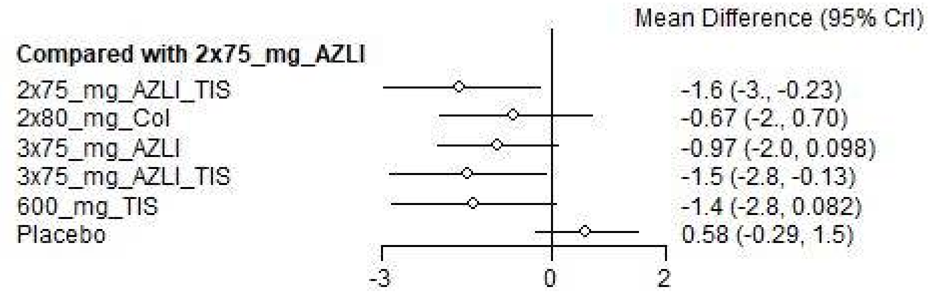

Figure S18. Forest plot for change in Pseudomonas sputum density interventions compared to 2 × 75 mg aztronam-lysine.

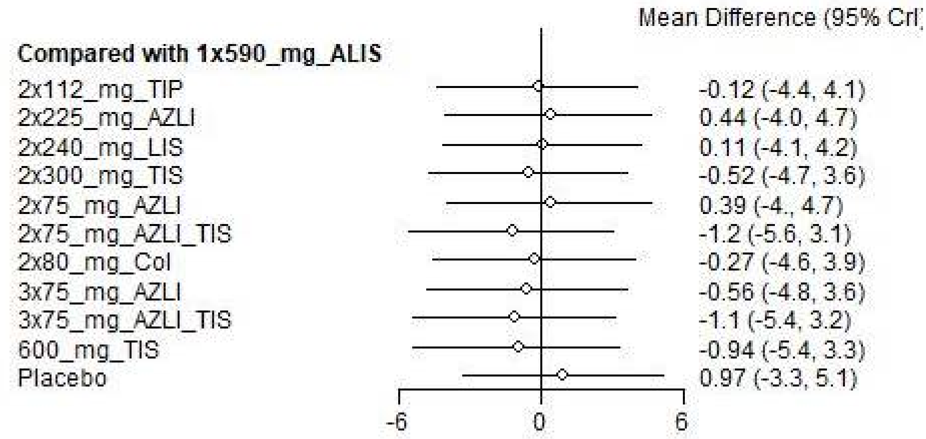

Figure S19. Forest plot for change in Pseudomonas sputum density interventions compared to 1 × 590 mg amikacin.

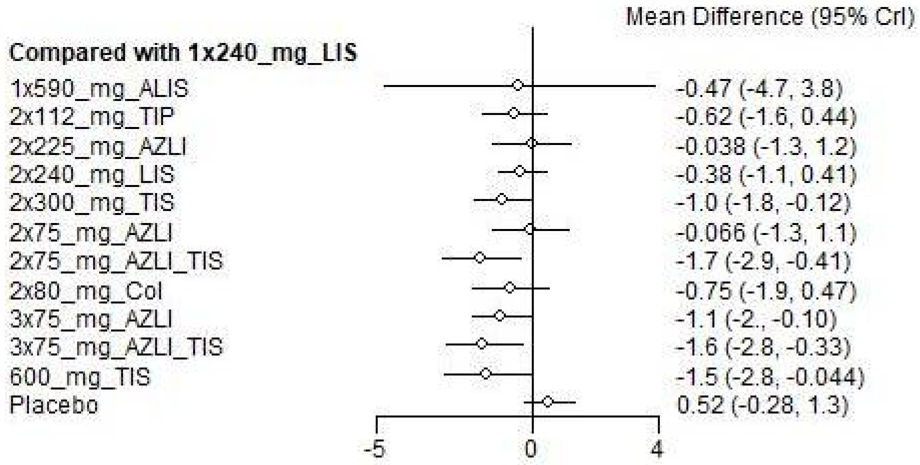

Figure S20. Forest plot for change in Pseudomonas sputum density interventions compared to 1 × 240 mg levofloxacin inhalation solution.

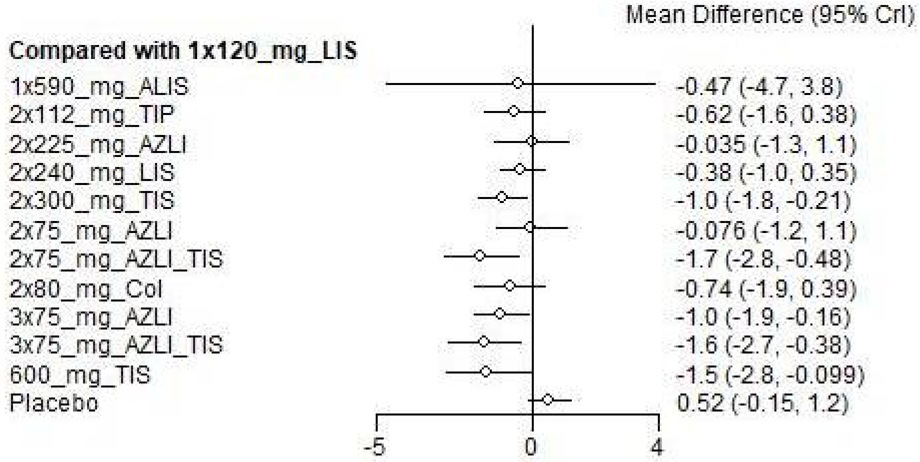

Figure S21. Forest plot for change in Pseudomonas sputum density interventions compared to 3 × 75 mg aztronam+run in tobramycin.

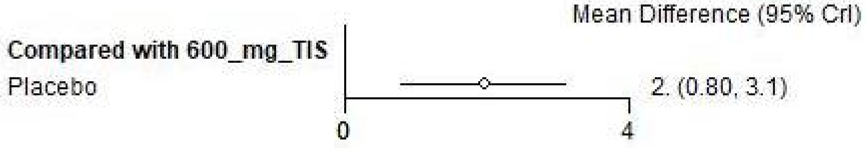

Figure S22. Forest plot for change in Pseudomonas sputum density interventions compared to 600 mg tobramycin.

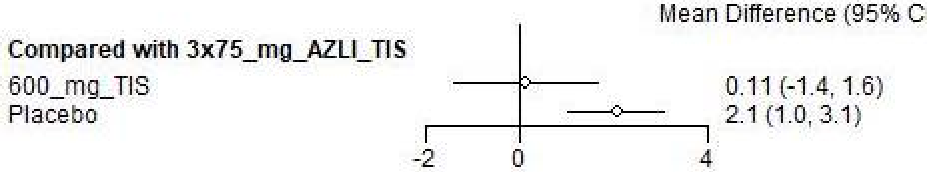

Figure S23. Forest plot for change in Pseudomonas sputum density interventions compared to 3 × 75 mg aztronam+run in tobramycin.

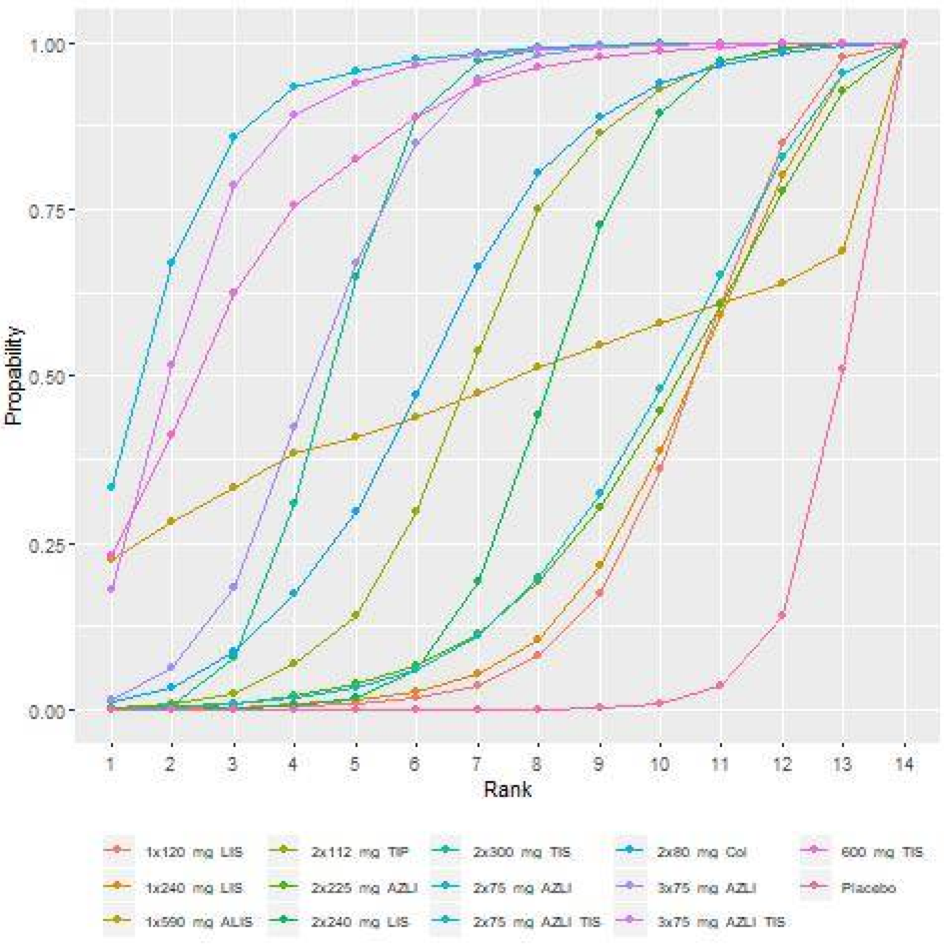

Figure S24. Cumulative ranking curves of change in Pseudomonas sputum density.

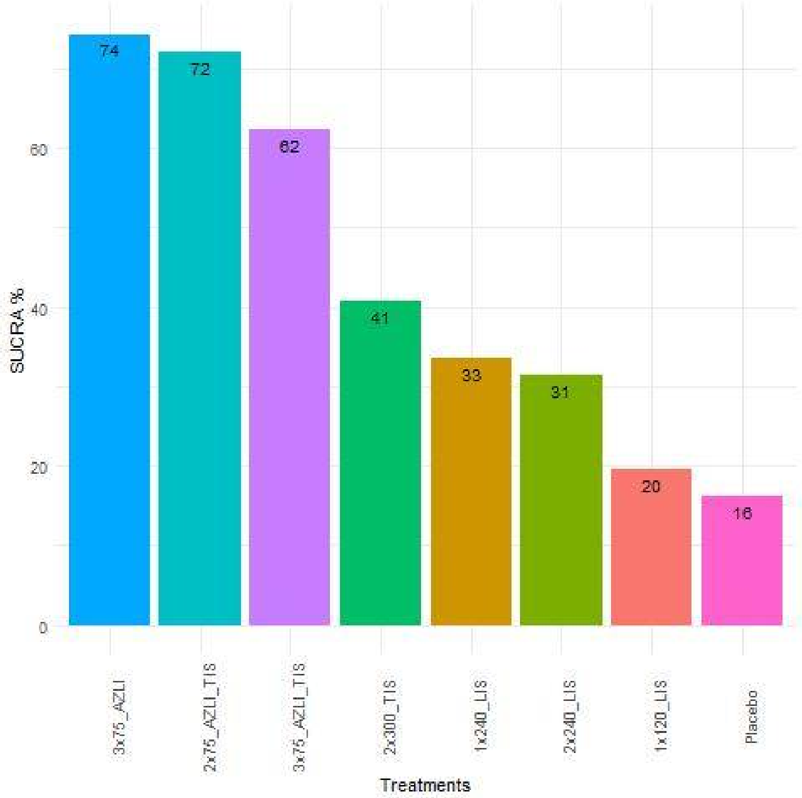

Figure S25. Surface under the cumulative ranking curves (SUCRA%) values of change in CFQR-RSS.

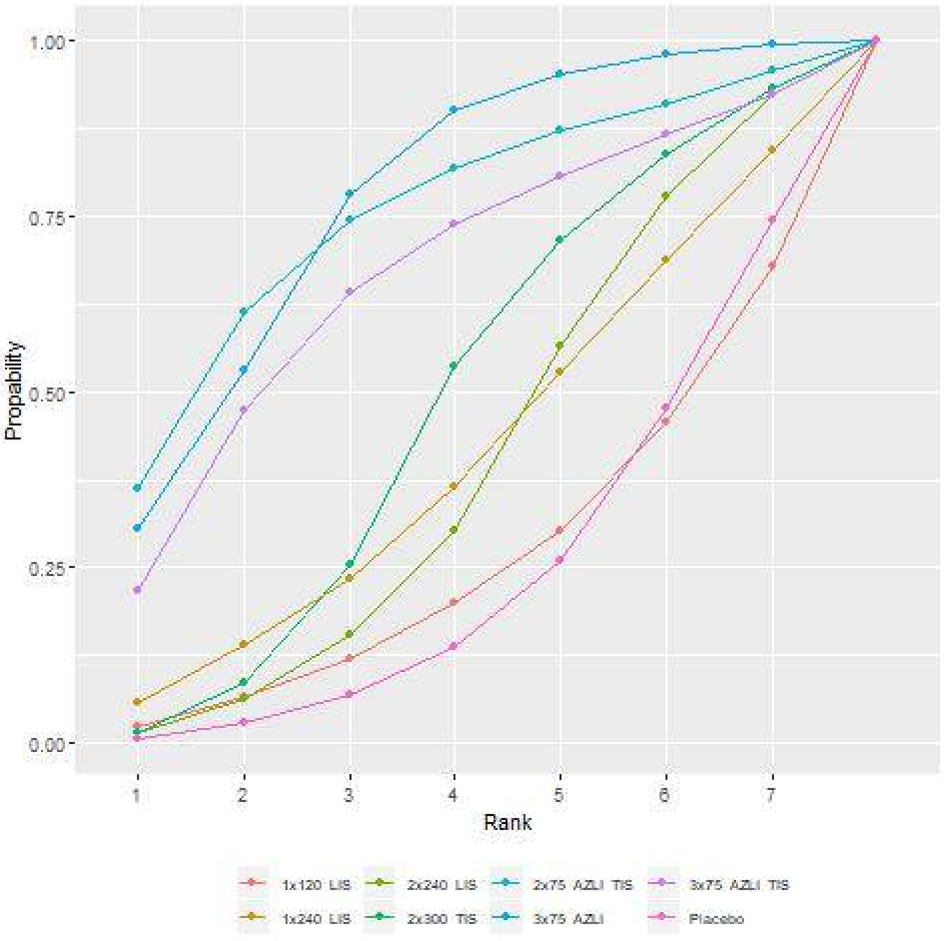

Figure S26. Cumulative ranking curves of change in CFQR-RSS.

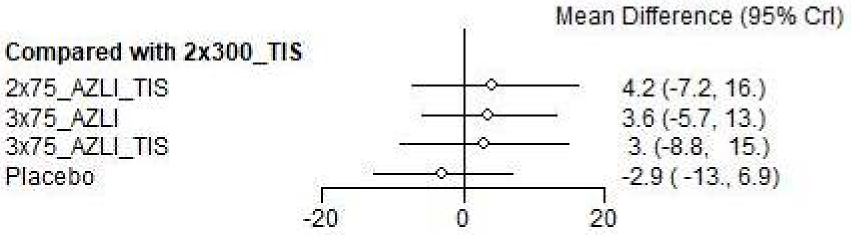

Figure S27. Forest plot for change in CFQR-RSS interventions compared to 2 × 300 mg TIS.

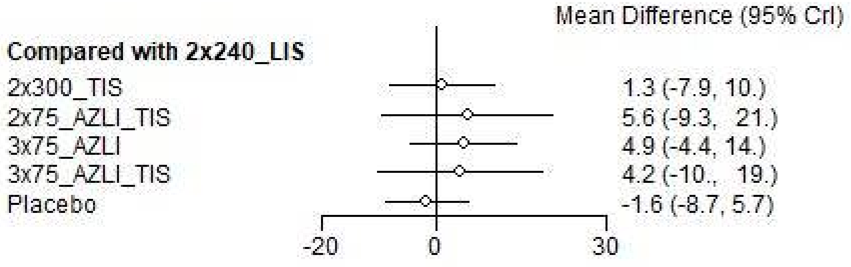

Figure S28. Forest plot for change in CFQR-RSS intervnetions compared to 2 × 240 mg LIS.

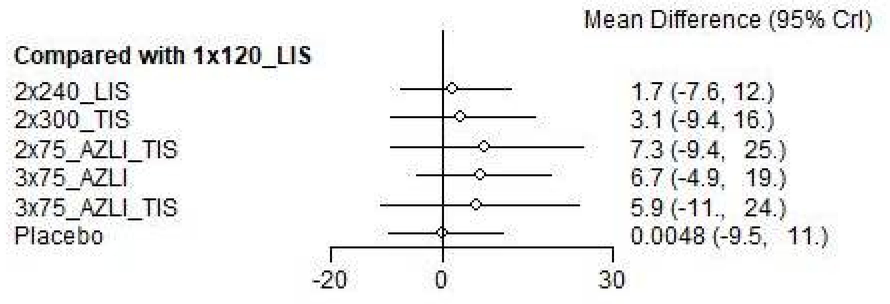

Figure S29. Forest plot for change in CFQR-RSS interventions compared to 1 × 120 mg LIS.

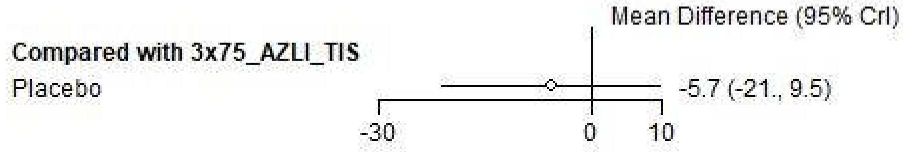

Figure S30. Forest plot for change in CFQR-RSS compared to 3 × 75 mg AZLI+TIS.

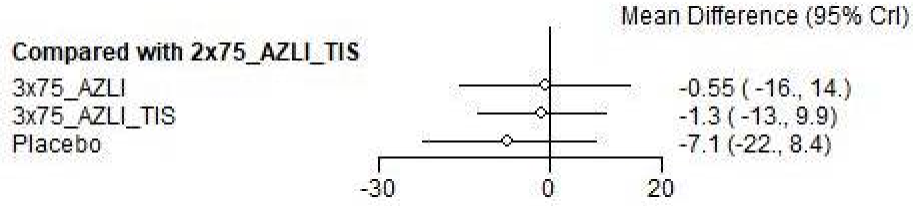

Figure S31. Forest plot for change in CFQR-RSS interventions compared to 2 × 75 mg AZLI+TIS.

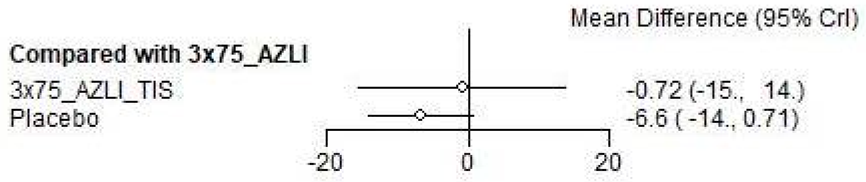

Figure S32. Forest plot for change in CFQR-RSS interventions compared to 3 × 75 mg AZLI.

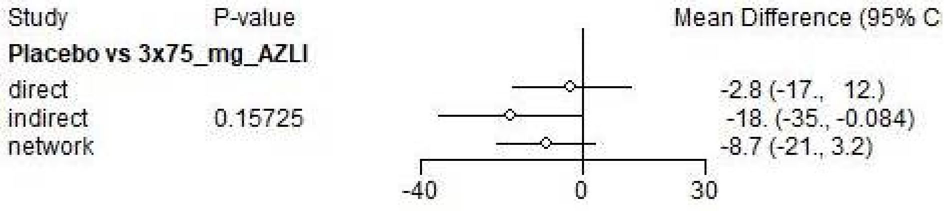

Figure S33. Consistency test in change in FEV1%\.

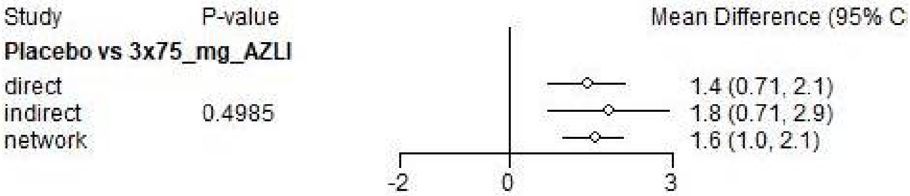

Figure S34. Consistency test in change Pseudomonas sputum density.

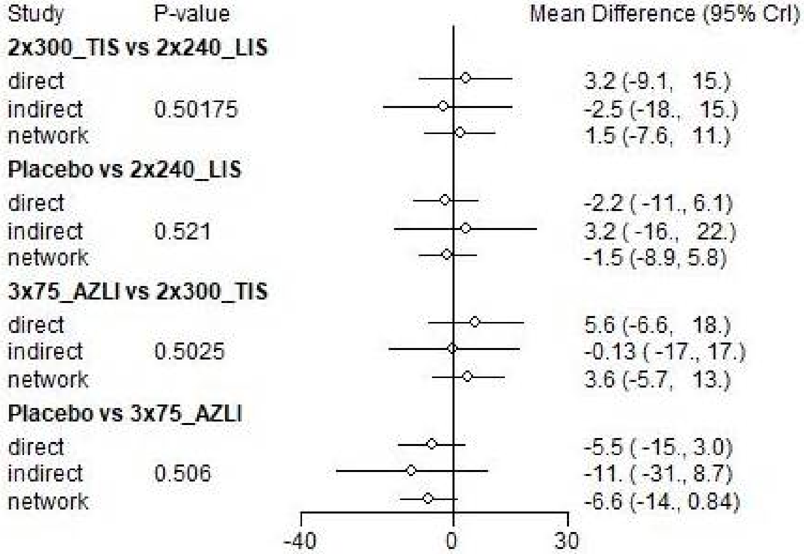

Figure S35. Consistency test in change in CFQR-RSS.

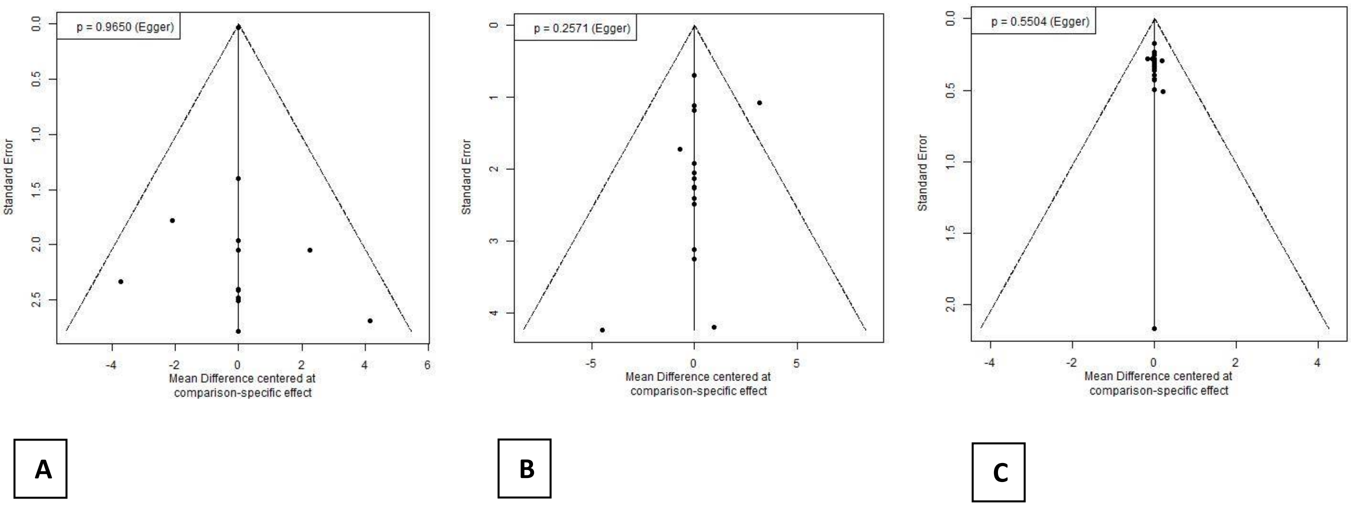

Figure S36. Funnel plot and Egger's test of A: change in FEV1%, B: change in Pseudomonas sputum density, C: change in CFQR-RS.

Assael	AZLI	TIS	FEV1%	1	?	?	+	+	?	!
Bilton D	ALIS	TIS	FEV1%	1	+	?	+	+	?	!
Flume P	LIS 240 mg	Placebo	FEV1%	1	?	+	+	+	?	!
GalevI	TIP	Placebo	FEV1%	1	+	+	+	+	?	!
Hodson	Tobramycin	Colistin	FEV1%	1	?	?	+	+	?	!
Geller D	levofloxacin	Placebo	FEV1%	1	?	+	+	+	?	!
Konstan M 2011	TIP	Placebo	FEV1%	1	?	+	+	+	?	!
Lenoir	TSI	Placebo	FEV1%	1	?	+	+	+	?	!
McCoy	AZLI BID	Placebo	FEV1%	1	?	+	+	+	?	!
Ramsey B	Tobramycin	Placebo	FEV1%	1	?	+	+	+	?	!
Ramsey B 1999	TIS	Placebo	FEV1%	1	?	+	+	+	?	!
Retsch-Bogart 2007	75 mg AZLI	Placebo	FEV1%	1	?	+	+	+	?	!
Retsch-Bogart 2009	75 mg AZLI	Placebo	FEV1%	1	?	+	?	+	?	!
Trapnel	FTI	Placebo	FEV1%	1	?	+	+	+	?	!
Wainwright	AZLI TID	Placebo	FEV1%	1	+	+	+	+	?	!
Konstan M 2010	TIP	TIS	FEV1%	1	?	?	+	+	?	!
Elborn	LIS	TIS	FEV1%	1	?	?	+	+	?	!

Figure S37. Summary of risk of bias assessment in change in FEV1%.

Retch-Bogart 2009.PA	AZLI	Placebo	Pseudomonas	1	?	+	+	+	?	!
Trapnel PA	FTI	FTI	Pseudomonas	1	?	+	+	+	?	!
McCoy PA	AZLI	Placebo	Pseudomonas	1	?	+	+	+	?	!
Assael PA	AZLI	TIS	Pseudmonas	1	?	+	+	+	?	!
Ramsey 1999 PA	LIS	Placebo	Pseudmonas	1	?	+	+	+	?	!
Geller PA	TIS	Placebo	Pseudomonas	1	?	+	+	+	?	!
Lenoir PA	LIS	TIS	Pseudomonas	1	?	+	+	+	?	!
Elborn PA	TIS	Placebo	Pseudomonas	1	?	?	?	+	?	!
Ramsey 93 PA	TIP	TIS	Pseudomonas	1	?	+	+	+	?	!
Konstan M 2010 PA	TIS	Placebo	Pseudomonas	1	?	+	+	+	?	!
Chuchalin PA	AZLI	Placebo	Pseudomonas	1	?	+	+	+	?	!
Retsch-Bogart 2007 PA	TIS	Colistin	Pseudomonas	1	?	+	+	+	?	!
Hodon PA	TIP	Placebo	Pseudomonas	1	?	+	+	+	?	!
Konstan 2011 PA	TIP	Placebo	Pseudomonas	1	?	+	+	+	?	!
Galeva PA	TIP	Placebo	Pseudomonas	1	?	+	+	+	?	!
Wainwright PA	AZLI	Placebo	Pseudomonas	1	+	+	?	+	?	!
Chuchalin	TIS	Placebo	Pseudomonas	1	?	?	+	+	?	!

Figure S38. Summary of risk of bias assessment in change in Pseudomonas sputum density.

Table S1. League table of change in FEV1%. The overall risk of bias assessment were judged to raise some concern and in line with the GRADE approach all comparisons were judged as very low quality ⊕◯◯◯.

3x75 mg AZLI+run-in Tob.												
0.41 (-16; 17)<br>⊕◯◯◯	2x75 mgAZLI+run-in Tob.											
4.1 (-15; 24)<br>⊕◯◯◯	3.7 (-16; 24)<br>⊕◯◯◯	2x112 mg TIP										
4 (-21; 29)<br>⊕◯◯◯	3.4 (-21; 28)<br>⊕◯◯◯	0.074 (-19; 19)<br>⊕◯◯◯	1x600 mg Tobramycin									
4.9 (-15; 25)<br>⊕◯◯◯	4.5 (-16; 24)<br>⊕◯◯◯	0.82 (-14; 15)<br>⊕◯◯◯	0.91 (-20; 21)<br>⊕◯◯◯	3x75 mg AZLI								
6.5 (-9.8; 23)<br>⊕◯◯◯	6 (-10; 22)<br>⊕◯◯◯	2.3 (-9.6; 13)<br>⊕◯◯◯	2.4 (-17; 21)<br>⊕◯◯◯	1.6 (-11; 14)<br>⊕◯◯◯	2x300 mg TIS							
6.4 (-8; 31)<br>⊕◯◯◯	6.1 (-18; 30)<br>⊕◯◯◯	9,7 (-0.84; 20)<br>⊕◯◯◯	2.4 (-21; 26)<br>⊕◯◯◯	1.5 (-18; 1)<br>⊕◯◯◯	0.12 (-18; 18)<br>⊕◯◯◯	80/20 mg FTI						
7.5 (-17; 32)<br>⊕◯◯◯	7.2 (-17; 31)<br>⊕◯◯◯	3.6 (-16; 22)<br>⊕◯◯◯	3.6 (-20; 27)<br>⊕◯◯◯	2.7 (-18; 24)<br>⊕◯◯◯	1.2 (-17; 20)<br>⊕◯◯◯	1.1 (-16; 18)<br>⊕◯◯◯	160/40 mg FTI					
8.5 (-12; 29)<br>⊕◯◯◯	7.9 (-13; 28)<br>⊕◯◯◯	4.4 (-11; 19)<br>⊕◯◯◯	4.4 (-16; 25)<br>⊕◯◯◯	3.4 (-13; 20)<br>⊕◯◯◯	2.3 (-9.6; 13)<br>⊕◯◯◯	2.0 (-18; 23)<br>⊕◯◯◯	0.73 (-20; 21)<br>⊕◯◯◯	2x240 mg Levofloxacin				
13 (-12; 37)<br>⊕◯◯◯	12 (-11; -35)<br>⊕◯◯◯	8.7 (-13; 28)<br>⊕◯◯◯	8.8 (-16; 33)<br>⊕◯◯◯	7.9 (-13; 29)<br>⊕◯◯◯	6.4 (-11; 23)<br>⊕◯◯◯	6.2 (-18; 31)<br>⊕◯◯◯	5.0 (-21; 29)<br>⊕◯◯◯	4.3 (-17; 25)<br>⊕◯◯◯	2x80 mg Colistin			
13 (-11; 39)<br>⊕◯◯◯	13 (-11; 38)<br>⊕◯◯◯	9.4 (-9.6; 29)<br>⊕◯◯◯	9.5 (-14; 33)<br>⊕◯◯◯	8.5 (-12; 30)<br>⊕◯◯◯	7.0 (-11; 26)<br>⊕◯◯◯	7.0 (-16; 30)<br>⊕◯◯◯	5.9 (-17; 29)<br>⊕◯◯◯	5.1 (-15; 26)<br>⊕◯◯◯	0.79 (-24; 25)<br>⊕◯◯◯	2x225 mg AZLI		
14 (-10; 38)<br>⊕◯◯◯	13 (-11; 39)<br>⊕◯◯◯	3.7 (-16; 24)<br>⊕◯◯◯	9.6 (-13; 33)<br>⊕◯◯◯	8.9 (-12; 30)<br>⊕◯◯◯	7.3 (-26; 11)<br>⊕◯◯◯	7.3 (-16; 30)<br>⊕◯◯◯	6.2 (-17; 29)<br>⊕◯◯◯	5.3 (-15; 25)<br>⊕◯◯◯	1.0 (-26; 24)<br>⊕◯◯◯	0.086 (-17; 17)<br>⊕◯◯◯	2x75 mg AZLI	
14 (-4.5; 32)<br>⊕◯◯◯	13 (-4.7; 32)<br>⊕◯◯◯	9.7 (-0.94; 20)<br>⊕◯◯◯	9.8 (-6.8; 26)<br>⊕◯◯◯	8.8 (-3.1; 21)<br>⊕◯◯◯	7.3 (-1.1; 16)<br>⊕◯◯◯	7.3 (-9.0; 24)<br>⊕◯◯◯	6.1 (-10; 23)<br>⊕◯◯◯	5.3 (-6.2; 18)<br>⊕◯◯◯◯◯	0.92 (-17; 20)<br>⊕◯◯◯	0.33 (-16; 16)<br>⊕◯◯◯	0.11 (-16; 16)<br>⊕◯◯◯	Placebo

Table S2. League table of change in CFQR-RSS.

**3x75 mg Aztreonam-lysine**							
**0.55 (-14; 16)**	**2x75 mg Aztreonam-lysine+run in Tob.**						
**0.72 (-14; 15)**	**1.3 (-9.9; 13)**	**3x75 mg Aztreonam-lysine+run in Tob.**					
**3 (-8.8; 15)**	**4.2 (-7.2; 16)**	**3.6 (-5.7; 13)**	**2x300 mg Tobramycin inhalation solution**				
**5.0 ('-6.5; 17)**	**5.6 (-11; 23)**	**4.2 (-13; 22)**	**1.4 (-11; 14)**	**1x240 mg Levofloxacin inhalation solution**			
**4.2 (-10; 19)**	**5.6 (-9.3; 21)**	**4.9 (-4.4; 14)**	**1.3 (-7.9; 10)**	**0.043 ('-9.8; 11)**	**2x240 mg Levofloxacin inhalation solution**		
**6.7 (-4.9; 19)**	**7.3 (-9.4; 25)**	**5.9 (-11; 24)**	**3.1 (-9.4; 16)**	**1.7 (-13; 9.1)**	**1.7 (-12; 7.6)**	**1x120 mg Levofloxacin inhalation solution**	
**6.6 (-0.74; 14)**	**7.1 (-8.4; 22)**	**5.7 (-9.5; 21)**	**2.9 (-6.9; 13)**	**1.5 (-11; 9.1)**	**1.6 (-5.7; 8.7)**	**0.0048 (-9.5; 11)**	**Placebo**

Table S3. summary of findings of change in *Pseudomonas* sputum density.

	TIS vs. TIP	TIS vs. 3 × 75mg AZLI TIS	TIS vs. 3 × 75 mg AZLI+TIS	TIS vs. 3 × 75 AZLI	TIS vs. 2 × 75 mg AZLI	TIS vs. Col	TIS vs. 2 × 240 mg LIS	TIS vs. Placebo	3 × 75 mg AZLI vs. Placebo	2 × 75 mg AZLI vs. Placebo	600 mg TIS vs. Placebo	TIS vs. ALIS
**Study limitations [1]**	↓	↓	↓	↓	↓	↓	↓	↓	↓	↓	↓	↓
-	-	-	some concerns	some concerns	some concerns	some concerns	some concerns	some concerns	some concerns	some concerns	some concerns	some concerns
⊕⊕⊕○ Moderate evidence	⊕⊕⊕○ Moderate evidence	⊕○○○ Very low evidence	-	↓↓	↓↓	↓↓	↓↓	↓↓	↓↓	↓↓	↓↓	↓↓
**Comments**	No significant difference	-	-	No significant difference	No significant difference	No significant difference	No significant difference	No significant difference	No significant difference	No significant difference	No significant difference	No significant difference
**Inconsistency [2]**	-	-	-	-	-	-	-	-	-	-	-	-
**Indirectness [3]**	-	-	-	-	-	-	-	-	-	-	-	-
**Publication bias [4]**	-				-	-	-	-	-	-	-	-
**GRADE**	⊕○○○ Very low evidence				⊕○○○ Very low evidence	⊕○○○ Very low evidence	⊕○○○ Very low evidence	⊕○○○ Very low evidence	⊕○○○ Very low evidence	⊕○○○ Very low evidence	⊕○○○ Very low evidence	⊕○○○ Very low evidence

[1] Information detailed in Figure S26. [2] Inconsistency was evaluated by node splitting did not suggest inconsistency. [3] Where there was only one head-to-head trial indirectness could not be judged. [4] Small study effect is unlikely to distort our results, as indicated by the funnel plots.

Table S4. summary of findings of change in FEV1%.

	TIS vs. TIP	TIS vs. 3 × 75 mg AZLI+TIS	TIS vs. 3 × 75 mg AZLI+TIS	TIS vs. 3 × 75 mg AZLI	TIS vs. 2 × 75 mg AZLI	TIS vs. Col	TIS vs. 2×240 mg LIS	TIS vs. Placebo	3 × 75 mg AZLI vs. Placebo	2 × 75 mg AZLI vs. Placebo	600 mg TIS vs. Placebo	FTI vs. Placebo
**Study limitations [1]**	↓	↓	↓	↓	↓	↓	↓	↓	↓	↓	↓	↓
**Comments**	some concerns	some concerns	some concerns	some concerns	some concerns	some concerns	some concerns	some concerns	some concerns	some concerns	some concerns	some concerns
**Imprecision**	↓↓	↓↓	↓↓	↓↓	↓↓	↓↓	↓↓	↓↓	↓↓	↓↓	↓↓	↓↓
**Comments**	No significant difference	No significant difference	No significant difference	No significant difference	No significant difference	No significant difference	No significant difference	No significant difference	No significant difference	No significant difference	No significant difference	No significant difference
**Inconsistency [2]**	-	-	-	-	-	-	-	-	-	-	-	-
**Indirectness [3]**	-	-	-	-	-	-	-	-	-	-	-	-
**Publication bias [4]**	-	-	-	-	-	-	-	-	-	-	-	-
**GRADE**	⊕◯◯◯ Very low evidence	⊕◯◯◯ Very low evidence	⊕◯◯◯ Very low evidence	⊕◯◯◯ Very low evidence	⊕◯◯◯ Very low evidence	⊕◯◯◯ Very low evidence	⊕◯◯◯ Very low evidence	⊕◯◯◯ Very low evidence	⊕◯◯◯ Very low evidence	⊕◯◯◯ Very low evidence	⊕◯◯◯ Very low evidence	⊕◯◯◯ Very low evidence

[1] Information detailed in Figure S10. [2] Inconsistency was evaluated by node splitting did not suggest inconsistency. [3] Where there was only one head-to-head trial indirectness could not be judged. [4] Small study effect is unlikely to distort our results, as indicated by the funnel plots.

1. Funding source

This study was funded by „GINOP-2.3.2-15-2016-00048 - STAY ALIVE" co-financed by the European Union (European Regional Development Fund) within the framework of Programme Széchenyi 2020, and by Human Resources Development Operational Programme Grant, Grant Number: EFOP 3.6.2-16-2017-00006 – LIVE LONGER which is co-financed by the European Union (European Regional Development Fund) within the framework of Programme Széchenyi 2020.Study funders had no role in the study design, data analysis, collection of data, interpretation of results, writing of the findings or in the decision of publication.

2. Quality of evidence

In line with the Revised Cochrane risk-of-bias tool for randomized trials (RoB 2) risk of bias assessment was completed on individual study-level. We selected the highest risk of bias individual study then we totalized the overall RoB-assessment of treatments. We downgraded the quality of evidence by two if the comparison was at high risk of bias, and downgraded it by one it was concluded to raise some concerns.

Inconsistency was evaluated by node splitting it did not suggest inconsistency for any outcome.

The population of the studies were clinically heterogenous with different baseline characteristics. The concealment of group allocations were different in the studies some of them were open label and and some of them were double blinded. Funnel plots were created for each outcome and Egger's tests were performed to assess small-study effect. Small study effect is unlikely to distort our results, as indicated by the funnel plots.
